# Parents’ Perspectives on Adaptive Sports in Children with Profound Intellectual and Multiple Disabilities

**DOI:** 10.3390/children8090815

**Published:** 2021-09-16

**Authors:** Marion C. Neyroud, Christopher J. Newman

**Affiliations:** 1 Faculty of Biology and Medicine, University of Lausanne, 1011 Lausanne, Switzerland; Marion.Neyroud@unil.ch; 2 Paediatric Neurology and Neurorehabilitation Unit, Lausanne University Hospital and University of Lausanne, 1011 Lausanne, Switzerland

**Keywords:** PIMD, physical activities, adaptive sports, children, disability

## Abstract

Children with profound intellectual and multiple disabilities (PIMD) need adaptations to participate in sports and it is more difficult for them to access these activities. We investigated the effects of adaptive sports in children with PIMD as perceived by their parents. The parents answered a postal questionnaire exploring the effects of adaptive sports during the 3 days following an activity. The questionnaire explored twelve domains of children’s daily lives, such as sleep and appetite. We calculated a composite score, including all of these domains, to assess whether the children globally benefited from adaptive sports. Of the families, 27/63 responded (participation 42.9%). Four domains improved after the sports activity in an important proportion of children (improvement in 64.0% of children for wellbeing, 57.6% for mood, 56.0% for comfort and 48.1% for sleep). Among the majority of children, the other eight domains remained mostly stable. Three quarters of parents reported a globally positive effect of adapted physical activities on their child. These findings support the further development and provision of adaptive sports for children with severe neurological impairments.

## 1. Introduction

Children with motor disabilities have difficulties accessing sports and participating in them [1]. They require adaptations that increase with the severity of their motor impairment and with the presence of co-morbidities. The amount of physical activity practised by these children, who lead a much more sedentary lifestyle than other children, is significantly lower than the usual recommendations and time spent exercising for children without disabilities [2]. Previous studies [1,3,4] have mainly explored the limitations and facilitators of accessing physical activities for children with a physical disability. These quantitative or mixed methods studies included perspectives from a variety of stakeholders (children, parents, physicians, therapists, sports and recreation industry personnel). One of the studies states that sport contributes to these children’s wellbeing, improves social and motor skills and reduces co-morbidities [4]; however, these associations are hypothetical. The effects of adaptive sports on the physical health and the quality of life in children with a disability and, more specifically, those with profound intellectual and multiple disabilities (PIMD), are thus largely unexplored.

The key defining characteristic of children with PIMD is the association of a severe-to-profound intellectual disability with a severe neuromotor impairment. These individuals have little or no apparent understanding of verbal language, little or no symbolic interaction with objects and are highly dependent for all activities of daily life [5]. Often they have associated sensory impairments, behavioural issues and present complex healthcare needs due to medical comorbidities (pain, sleep disorders, musculoskeletal complications, digestive and respiratory issues) [6]. PIMD is estimated to be prevalent in between 0.4 and 1.3‰ of children [7,8] and its aetiology is variable, resulting from severe acquired or congenital neurological disorders, which namely include diagnoses such as cerebral palsy, genetic or metabolic encephalopathies and certain genetic syndromes [9].

Physical activity maintains or improves physical and mental health, fitness and participation in daily life. Children with PIMD present low levels of physical activity, use small fractions of their heart rate reserves [10] and have low levels of total energy expenditure [11] with potential adverse effects on their general health. Adaptive sports [12] for children with PIMD provide engagement in physical activities adapted to their function and aim to get them moving actively in a playful, supervised and secure context. These include group activities such as ball games (e.g., Boccia) [13] or aquatic training [14], as well as outdoor activities such as horseback riding [15] or tandem skiing [16]. Adaptive sports do not include physical therapies (physiotherapy, occupational therapy) or activities of daily living such as using a walker or standing up.

The aim of our study was to explore the effects of adaptive sports in children with PIMD. Our primary objective was to study their parents’ perceptions of these effects. Our secondary objective was to explore the potential associations between these effects and the severity of the motor disability.

## 2. Methods

### 2.1. Study Design

We conducted a cross-sectional descriptive study. The study was approved on 1 April 2020 by the Regional Ethics Committee (decision 2019-02351, CER-VD).

### 2.2. Selection Criteria

Participation in our study was open to the parents of children aged 4 to 19 years old with a diagnosis of PIMD who attended Lausanne University Hospital’s Paediatric Neurorehabilitation Clinic. We excluded parents whose level of French we deemed insufficient to complete the questionnaire confidently.

### 2.3. Questionnaire

The questionnaire was developed from the thematic analysis of semi-structured interviews conducted with a sample of stakeholders: a paediatric neurorehabilitation specialist, a childhood disability nurse, an adaptive sports teacher, a tandem ski instructor and a parent of a child with PIMD. These interviews focused on the challenges and benefits of adaptive sports for children with PIMD and allowed us to identify twelve domains of potential improvement (see below).

The questionnaire contained two sections: (1) general information on the parent(s) filling in the questionnaire and their child, (2) items specifying the effects of adaptive sports on their child.

In the first section we asked for the gender and age of the parents and child, and whether the questionnaire was completed by one parent or both. We included a question on the adaptive sports practised by the child, as well as their frequency and the context in which they took place (at school, with the family, in a sports club, etc.).

The second section explored the effects of adaptive sports on the child, and was divided into twelve domains: sleep, wakefulness, appetite, eating, communication, behaviour, attention, mood, wellbeing, comfort, movement and activity level. Parents were asked to report the effects of a self-selected activity by considering the three days following its completion. To achieve this, the person(s) completing the questionnaire classified the effect on a 5-level Likert scale with the responses “much better”, “better”, “as usual”, “worse” and “much worse”. Finally, parents were queried on the ideal frequency and duration of an adaptive sports activity.

The questionnaire script is available as a Supplementary File (Table S1).

### 2.4. Recruitment

During spring 2020, we invited parents of children with PIMD via postal mail to take part in the study by completing the attached questionnaire. A reminder was sent one month after the initial invitation and the deadline for participation was set at one month after receiving the reminder. This gave participants two months to complete and return the questionnaire with a signed consent form.

As participation in this study was voluntary, we provided all participants the opportunity to withdraw at any time, in accordance with the information they received in the invitation. Nevertheless, the data collected up to that point were analysed, so as not to compromise the value of the study as a whole.

### 2.5. Clinical Data

We collected the following data on the children from their medical records at Lausanne University Hospital: age of the child, gender, primary and secondary diagnoses, IQ or mental age estimate and mobility (walking with/without an assistive device/active mobility in wheelchair/passive mobility in wheelchair).

### 2.6. Statistics

Responses were reported by descriptive statistics with means and standard deviations for continuous values, and proportions and frequencies for categorical values. We reduced the 5-level Likert scale to three categories for analyses (improved, same and worse). We calculated a composite score by including all the items in order to assess whether the children globally benefited from adaptive sports activities or not. To achieve this, we added for each child the number of domains showing an improvement after the physical activity (to which we gave the value +1), to the number of domains remaining stable (to which we gave the value 0) and the number showing a negative effect (to which we gave the value −1). Associations were explored by cross-tabulations and Chi-square tests. We associated each domain with the child’s gender, age and mobility. Statistical significance was set at a Bonferroni corrected *p*-value of 0.0042 as we had twelve factors. We did not use statistical methods to infer missing data. We used SPSS Statistics v.25 (IBM, Armonk, NY, USA) to perform the analyses.

## 3. Results

### 3.1. General Information

We sent invitations to 63 families and received 27 questionnaires in return, with a participation rate of 42.9%. Among the 27 parents who responded, there were 22 women (81.5%) and 5 men (18.5%), their mean age was 44.1 years (SD 6.3). Eighteen of them completed the questionnaire alone, seven with the child’s other parent, and two did not provide this information. As for the children, we had a distribution of 16 girls (59.3%) and 11 boys (40.7%) and their mean age was 11.2 years (SD 4.7). All of them had a profound intellectual disability, twenty-one were passive wheelchair users, three were active wheelchair users and three walked with assistive devices.

All 27 (100%) children took part in swimming pool activities, most often with the guidance of an aquatic instructor, the use of flotation devices as required and an emphasis on individualised aquatic skills [17]. Eighteen (66.7%) children took part in adaptive skiing using professionally piloted tandem skiing devices [16]. Fourteen (51.9%) participated in a variety of school adaptive physical activity programmes. Ten children (37.0%) took part in horse riding, ten (37.0%) in adaptive sailing, eight (29.6%) in adaptive hiking using all-terrain one-wheeled chairs with the help of two guides [18], four (14.8%) in adaptive cycling on adaptive tricycles or adaptive tandem bikes, one (3.7%) in tandem paragliding and one (3.7%) in tandem paddling. Most children had had the opportunity to experience different types of adaptive sports. Fourteen children practised sports once a week or more (51.9%), six children more than once a month (22.2%) and four children more than once a year (14.8%); four parents did not provide information on the frequency of their child’s adaptive sports.

The majority of responses on the effects of adaptive sports were given regarding the participation in a swimming pool session (for 10 of the children), with others regarding tandem skiing (five), horseback riding (two), school adaptive gymnastics (one) or adaptive cycling (one); eight parents did not specify which of the adaptive sports they considered for their responses on effects.

### 3.2. Effects of Adaptive Sports

#### 3.2.1. Effects by Domain

**Figure 1:**
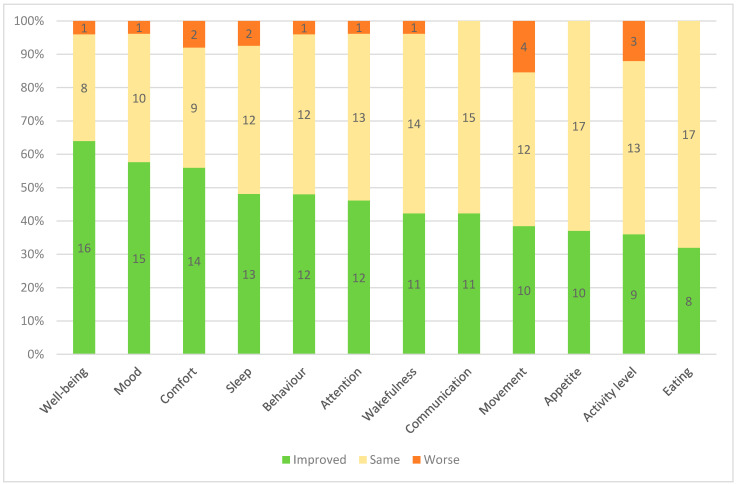
Stacked bar chart of the effects of adaptive sports on each domain ranked from the most to least positive effect.

The effects of adaptive sports per domain are summarized in Figure 1.

- Quality of life:

General wellbeing improved in 64.0% of children with PIMD, mood in 57.6% and comfort in 56.0%, with only one to two children experiencing a worsening wellbeing in these domains after an adaptive sports session.

- Mental functions:

After adaptive sports, the behaviour improved for 48.0% of children, attention improved for 46.2% and spontaneous communication improved for 42.3%.

- Sleep and arousal:

Sleep improved among 48.1% of children and wakefulness for 46.2% after an adaptive sports session. Only two children experienced a worsening of sleep.

- Appetite and eating:

Appetite improved in 37.0% of children, eating improved in 32.0% and none experienced a worsening of appetite.

- Spontaneous movement and activity:

Movement improved for 38.5% of children and 36.0% had a heightened activity level after adaptive sports; however 15.4% of children were reported as less mobile and 12.0% as less active after sports.

#### 3.2.2. Association Analyses

None of the analyses yielded significant associations between the effects in specific domains (sleep, wakefulness, appetite, eating, communication, behaviour, attention, mood, wellbeing, comfort, movement and activity level) or the composite score and, on the other hand, children’s personal factors (gender, age, mobility).

#### 3.2.3. Ideal Duration and Frequency of Sessions

Parents reported an adaptive sports session should ideally last 45 min (11 parents, 42%), half an hour (five parents, 19%), 1 hour (five parents, 19%), 2 h (four parents, 15%) or more (one parent, 4%). As for the ideal frequency of participation in an activity, 12 parents (46%) responded to more than one session per week, twelve parents (46%) to one session per week, one parent (4%) to one session every 2 weeks and one parent (4%) selected the category “other” with no further detail.

#### 3.2.4. Composite Score

**Figure 2:**
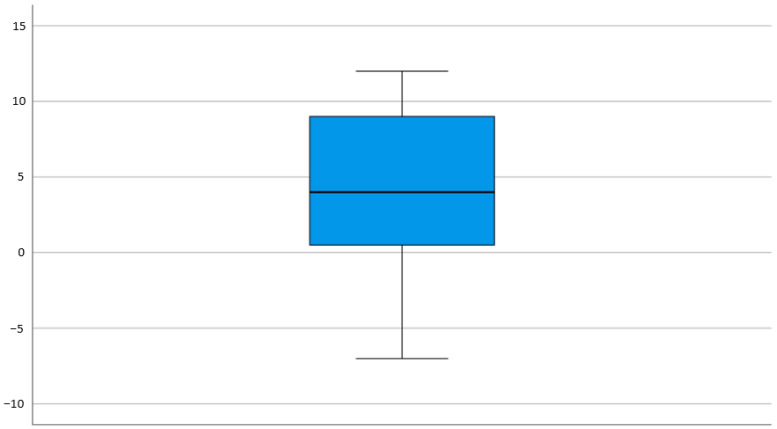
Box plot of the composite score of effects of adaptive sports in children with PIMD.

The composite score showed an overall beneficial effect of adaptive sports for twenty children (74.1%), a neutral effect for five children (18.5%) and two children (7.4%) experienced an overall negative effect (with one child accounting for negative effects in seven domains, the other for whom only decreased activity was reported). The median composite score was 4, with 25th and 75th centiles of 1 and 9, respectively (Figure 2).

## 4. Discussion

The aim of this study was to explore parents’ perspectives on the effects of adaptive sports in children with PIMD. We focused on parent opinions, since, for children with PIMD, who are strongly limited in their ability to communicate, parents are widely acknowledged by professionals and themselves as the best experts of their children’s experiences and needs [19]. These parents develop a heightened experiential and embodied knowledge of their child with PIMD, which provides them with privileged access to their child’s communication, expressions of wellbeing, or lack thereof, and pain.

Overall three quarters of parents reported a globally beneficial effect of adaptive sports for their child as seen in the composite score, with improvements on average in four of the explored domains.

The three domains that improved most (in more than half of the children), which we grouped under the concept of “quality of life”, were wellbeing, mood and comfort. Wellbeing, popularly often coined as “happiness” [20], improved for nearly two thirds of the children. Despite its subjective nature, wellbeing is a valid outcome that informed us of how parents thought their child was feeling, by holistically integrating both mental and physical health dimensions. Children and adolescents with disabilities who were able to self-report on their experience, previously highlighted the importance of the happiness and enjoyment provided by taking part in sports [21]. Likewise, in typically developing children and youth, psychological and social constructs that showed positive associations with sports participation included wellbeing, enjoyment, as well as mental health and perceived health [22]. Regarding mood, which more specifically reflects the mental component of wellbeing, previous literature demonstrated positive associations with sports in populations with and without disabilities. In typically developing children, several studies demonstrated the protective effect of increased physical activity against depressive and anxiety symptoms throughout school age and adolescence [23,24,25]. In adults with severe neurological impairments the practice of Boccia demonstrated positive influences on psychological health and mood [13] while young people with disabilities who practised sports reported improvements in confidence and self-esteem [21]. Children with PIMD have numerous reasons to experience discomfort and pain, and in our study adaptive sports seemed to be able to provide relief and improved comfort (reflecting the physical component of wellbeing) for a majority of children. Sedentary behaviour and low participation in physical activity were reported as risk factors for chronic pain, especially musculoskeletal pain, in typically developing children and adolescents [26,27,28], highlighting the value of moderate-to-vigorous physical activity in preventing these symptoms. Beyond their sedentary behaviour, certain children with PIMD present high degrees of physical disuse, which is strongly associated with acute and chronic pain via mechanical hyperalgesia, with peripheral and central sensitization [29]. Even more than in typically developing children, regular movement and physical activity such as those provided by adaptive sports may play an important role in supporting comfort and preventing pain.

Mental functions, namely behaviour, communication and attention improved in a non-negligible 40 to 50% of children with PIMD. If it is impossible to ascertain whether these improvements were secondary to the general improvement of wellbeing or conversely, these improvements all have a prosocial value for children whose baseline interactions are strongly limited. Physical activity interventions in typically developing youngsters positively affect cognition, namely executive functions and, more specifically, attention, knowing that a single bout of physical activity increases cerebral blood flow and the secretion of neurotransmitters, leading to increased levels of arousal and attention [30]. Similar phenomena may be at play in children with PIMD.

Adaptive sports improved sleep and wakefulness in above 45% of children with PIMD. Overall physical activity interventions seem to have a positive effect on sleep in the general population [31] with positive influences of regular physical activity on sleep latency, duration and quality [32]. Improving sleep can alone prove a worthwhile goal of adaptive sports in children with PIMD, given the high frequency of sleep disorders in this population and the spiralling negative effects of sleep deprivation on the children’s and families’ health and wellbeing [33]. Recent guidelines have emphasised the importance of taking into account both physical activity and sleep around-the-clock to improve the health and quality of life outcomes of children with neurological disabilities [34].

Among physical outcomes, appetite and eating improved in more than a third of children with PIMD, and no negative effects were reported. A number of the included children had predominantly enteral feeding and parents would presumably not have reported any change for them. For those who predominantly ate orally, it seems likely that the increase in energy expenditure associated with adaptive sports (even if possibly limited in this population), combined with the positive effects reported on wakefulness, attention and spontaneous activity and movements in the follow-up to the activity, contributed to their positive outcome. Again, adaptive sports could benefit the general health of children with PIMD, knowing that they are prone to undernutrition [9].

While more than a third of parents reported spontaneous movements and global activity levels increased after an adaptive sports session for their child, a significant minority of 12 to 15% reported contrary results, with decreases in movement and activity. For the latter parents, comments within the questionnaire attributed these decreases to exertional fatigue after the physical activity session or to a decrease in agitation, and conversely to what we had hypothesised, most of these parents did not consider these effects as negative. It seems likely that the post-sports motor and activity levels of children with PIMD are highly individual, depending on the individual characteristics of the child (such as motor abilities and spontaneous behaviour and personality), as well as on the type and intensity of the adaptive sports activity, with certain sports potentially more relaxing (e.g., swimming pool activities) than others (e.g., adaptive skiing).

We conducted a limited number of association analyses and none were significant. Children with PIMD conceivably draw the same benefits from adaptive sports, irrespective of age or gender. For baseline motor ability it is possible that, with the strong predominance of passive wheelchair users and the small groups of more active children, we lacked the statistical power to infer potential associations.

Regarding the provision of adaptive sports, a large majority of parents supported that their child take part in adaptive sports once a week or more and, on average, 45 min-sessions seemed the most suitable. In our study just over half the children took part in adaptive sports, at least on a weekly basis. Current World Health Organisation recommendations are that children and adolescents should complete at least an average of 60 min per day of moderate-to-vigorous intensity physical activity, across the week and that they should take part in vigorous-intensity aerobic activities, as well as those that strengthen muscle and bone at least 3 days per week [35]. For all populations, the benefits of physical activity and limiting sedentary behaviour outweigh the potential harms. Globally, it is estimated that less than 20% of adolescents reach these goals [36] and the presence of a disability further decreases the likelihood of attaining them. Realistically, children with PIMD cannot attain these goals due to their high level of dependence and restricted motor and cognitive abilities. However, the recommendations also acknowledge that some physical activity is better than none, and support that all individuals start with small amounts of physical activity and gradually increase frequency, intensity and duration over time within their possibilities. In free comments, several parents reported that adaptive sports had more impact on their child when practised more regularly and in short bursts. It is likely that children with PIMD become more fatigued during long sessions with the risk of reducing the beneficial effects of the activity. Conversely, they may not fully benefit from the potential positive effects if the sports sessions are too far apart.

Our study presented a number of limitations. The strongest limitation was that our sample size was limited and that our population was concentrated within a small region (canton Vaud, Switzerland, ca. 800,000 inhabitants), potentially decreasing the generalizability of our findings and our ability to infer associations. With a participation rate below 50% a participation bias cannot be excluded, possibly in favour of those families whose children take part in adaptive sports; however, there were no statistically significant differences between responders and non-responders in terms of the age or gender of their children (we were unable to control for other factors). Parents as hetero-evaluators integrated a subjective judgement to their responses, but, as mentioned previously, parents of children with PIMD are consensually considered as the most expert assessors of their children. This was a descriptive, non-controlled study, so inferences about causality between adaptive sports and observed effects were limited. Finally, since the effects of adaptive sports were reported on the basis of a parent-selected previous activity a degree of recall bias is possible.

Further prospective studies would be useful to confirm our findings by the direct observation of the effects of adaptive sports both during and after the activity. These should include the use psychometrically valid tools for the hetero-evaluation of certain impacted domains, such as those pertaining to quality of life, pain or communication. Additionally, the use of wearable sensors for these studies could allow for the collection of quantitative and objective data, not only on movement, physical activity and heartrate during adaptive sports, as previously demonstrated, in children with PIMD during tandem skiing [16], but also around-the-clock with longer term monitoring, potentially including sleep measurements [37].

## 5. Conclusions

In this first study of the effects of adaptive sports in children with PIMD we demonstrated that, as the best experts of their child, parents largely reported positive outcomes, both physically and mentally. Beyond these individual outcomes, adaptive sports provided the opportunity for these children and their families to engage socially and in meaningful activities, while breaking down barriers to participation, raising awareness and decreasing negative attitudes. Further initiatives are warranted to explore the effects of regular inclusive sports in children with PIMD and to improve these children’s access to these activities, in line with fundamental children’s rights and World Health Organisation goals.

## Data Availability

The data presented in this study are available on request from the corresponding author.

## Supplementary Materials

The following are available online at www.mdpi.com/2227-9067/8/9/815/s1, Table S1: Questionnaire script.
