# Supplementary material for: Parents’ Perspectives on Adaptive Sports in Children with Profound Intellectual and Multiple Disabilities
Source: Children (Basel). 2021 Sep 16;8(9):815. doi: 10.3390/children8090815 (PMC8471413; doi:10.3390/children8090815)
Supplement: Supplementary file 1 [file children-08-00815-s001.zip › children-1355382-supplementary.pdf]

Table S1 Survey script (translated from the original French version)

Effects of adaptive sports activities in children with multiple disabilities - a descriptive study of parents' perspectives

Please answer each of the following questions as accurately as possible by considering the three days following an adaptive sports activity in which your child participated.
If your child participates or has participated in more than one activity, please choose the one you think benefits your child most.
By adaptive sports, we mean organized physical activities adapted to children with severe physical disabilities. These include activities that take place in a playful and supervised context, such as group and indoor activities (e.g. Boccia) or outdoor activities such as horseback riding or tandem skiing. These activities do not include therapies (physiotherapy, occupational therapy, psychomotor therapy), nor activities of daily living (such as the use of a walker or standing).
We are interested in knowing what effects of physical activity you observe in the days following the activity. There are no correct or incorrect answers.
Your answers are confidential. Thank you very much for your participation.

1. You are:
   ☐ a woman
   ☐ a man

2. You are ____ years old.

3. You are filling in this questionnaire:
   ☐ alone
   ☐ with the child's other parent

4. 4. Your child is:
   ☐ a girl
   ☐ a boy

5. Your child is ____ years old

6. In which adaptive sports has your child taken part ?
   Please tick these activities and indicate how often he/she participates in them (less than once a year, once or more than once a year, once or more than once a month, once or more than once a week) and the context in which he/she practices this sport (school, family, club or association, other).

   Table including : Swimming pool activity / Horseback riding / School physical activity programme / Adaptive skiing / Rafroball (a regional variant of Boccia) / Adaptive hiking / Adaptive sailing / Other – specify __________________

   If your child participates in more than one activity, please specify which one sports you choose to further complete the questionnaire: __________________

   My child's sleep and wakefulness
   After a session of adaptive sports ...
7. ... my child sleeps at night — Much better / Better / As usual / Worse / Much worse
8. ... my child’s daytime wakefulness is — Much better / Better / As usual / Worse / Much worse
   Comments : ____

   My child's appetite and eating
   After a session of adaptive sports ...
9. ... my child's appetite is — Much better / Better / As usual / Worse / Much worse
10. ... my child eats — Much better / Better / As usual / Worse / Much worse
   Comments : ____

My child's mental function

After a session of adaptive sports ...

11. ... my child communicates — Much better / Better / As usual / Worse / Much worse
12. ... my child behaves — Much better / Better / As usual / Worse / Much worse
13. … my child’s attention is — Much better / Better / As usual / Worse / Much worse

Comments : ____

My child's well-being

After a session of adaptive sports ...

14. ... my child's mood is — Much better / Better / As usual / Worse / Much worse
15. ... my child's comfort is — Much better / Better / As usual / Worse / Much worse
16. ... my child's well-being is — Much better / Better / As usual / Worse / Much worse

Comments : ____

My child's activity

After a session of adaptive sports ...

17. ... my child moves — Much better / Better / As usual / Worse / Much worse
18. ... my child’s spontaneous activity is — Much better / Better / As usual / Worse / Much worse

Comments : ____

19. Are there any other effects that you have experienced as a result of physical activity that we have not covered in this questionnaire?
    ☐ Yes
    ☐ No

20. If yes which ones? ____

21. For a physical activity session to be good for your child, he or she should ideally benefit from it:
    ☐ more than once a week (specify: ___________)
    ☐ once a week
    ☐ once every fortnight
    ☐ once a month
    ☐ other : ____________

22. How long should the activity last to be most beneficial?
    ☐ less than 30 minutes (specify: ___________)
    ☐ 30 minutes
    ☐ 45 minutes
    ☐ 1 hour
    ☐ 2 hours
    ☐ more than 2 hours (specify: ___________)

23. Final comments: ____
